# Podcasting as a tool to take conservation education online

**DOI:** 10.1002/ece3.7353

**Published:** 2021-03-23

**Authors:** Bronson K. Strickland, Jarred M. Brooke, Mitchell T. Zischke, Marcus A. Lashley

**Affiliations:** ^1^ Department of Wildlife, Fisheries, and Aquaculture Mississippi State University Mississippi State MS USA; ^2^ Department of Forestry and Natural Resources Purdue University West Lafayette IN USA; ^3^ Department of Wildlife Ecology and Conservation University of Florida Gainesville FL USA

**Keywords:** extension, higher education, online pedagogy, podcast

## Abstract

Traditional forms of higher learning include teaching in the classroom on college campuses and in‐person adult‐focused public outreach events for non‐students. Online college degree programs and public outreach platforms have been steadily emerging, and the COVID‐19 pandemic has, at least temporarily, forced all related ecology and evolutionary biology programs to move to online delivery. Podcasting is a form of online mass communication that is rapidly gaining popularity and has the flexibility to be incorporated into the pedagogical toolbox for the online classroom and remote public outreach programming. Podcasting is also becoming more popular in the ecology and evolutionary biology field. Here, we describe the great potential of podcasting to transform the learning experience, present a case study of success from the United States, provide a table of podcast recommended by ecologist responding to a listserv, and provide a road map for adoption and utilization of podcasting for the future.

## Graphical abstract

Podcasting is easy to produce and highly versatile in the types of content that can be delivered. Here, we provide a list of podcast labels related to ecology, provide tips on recording our own podcast, and provide tips and an example for using podcast to deliver conservation education.

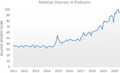

## 1 INTRODUCTION

**FIGURE 1:**
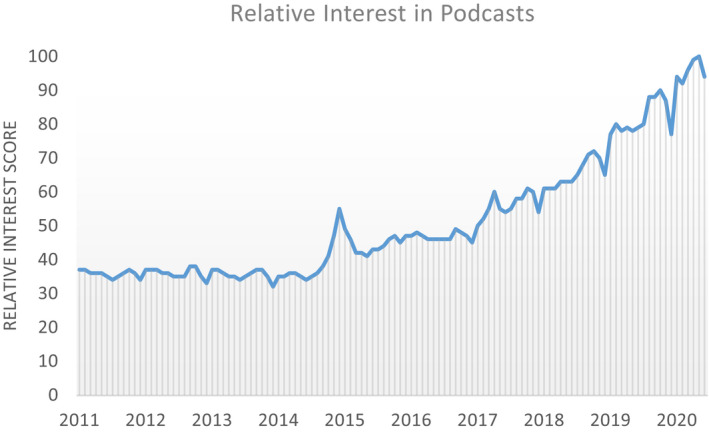
Global google trends data for the term podcast show a steadily increasing relative interest over the last decade (data‐autogenerated for the term “podcast” in google trends at https://trends.google.com/trends/). Google trends estimate the adjusted proportion of search volume for a particular term (i.e., relative interest) which can provide a useful indicator of changes in behavior or interest in topics over time based on searches for the term in the google search engine

Today, information is available like no other time in history. The general public and students alike can get information from traditional program delivery methods like classroom lectures, laboratories, field courses, workshops, seminars, and field days, but also from magazine articles, websites, and online videos. The term podcast, a combination of the words “iPod” and “broadcast” (Hendrickson et al. 2010), is defined as “a digital audio file created and then uploaded to an online platform to share with others” (Phillips, 2017). A major advantage of podcasts is that they allow audiences to listen to educational content asynchronously while engaged in other activities like driving, exercising, or working in the yard. According to google trends, which can be an effective indicator of the change in relative interest in a topic by global populations (Vosen & Schmidt, 2011), search volume for the term “podcast” has been increasing in relative interest (Figure 1). Other trend in society also indicates that podcasting is increasing in popularity. For example, according to Edison Research (2017), 60% of people aged 12 and older were aware of the term “Podcasting,” in 2017 as compared to only 22% in 2006. Also by 2017, 40% of the respondents had listened to a podcast, and 24% listened to podcasts monthly. Of the respondents that listened monthly, 77% of listeners are between the ages of 18 and 54, and another 16% were aged 55 or greater, thus a broad range of age demographics are regularly listening. Edison Research (2019) reported in the United States by 2019, 32% of people ≥12 years of age listened to a podcast in the last month, up from 9% in 2008. What is more, 22% of Americans ≥12 years of age are weekly podcast listeners, as compared to 7% in 2013 (Edison Research, 2019). The Apple Podcasts service alone features more than 1.2 million podcasts with over 32 million episodes (Lewis, 2020), and in 2018, there were 50 billion podcast downloads (Locker, 2018). Based on these studies from the United States, this communication platform appears to be an increasingly popular medium for communication.

Podcasting may also be a viable instrument for disseminating educational information to both adult and youth audiences as 74% of survey respondents said they listened to podcasts “to learn new things” (Edison Research, 2019). Along with the convenience of listening “on the go,” podcasts are popular because they can be experienced à la carte by connecting audiences with specific topics. A salient opportunity and challenge with this platform, and many modern communication channels, are quality control. That is, most anyone can record content and reach an audience to share their ideas and perspectives. When these ideas and perspectives are science‐based, the platform reaches new audiences and can have important impacts in society. When the voices elevated through the platform are not science‐based, the platform presents challenges of misinformation. Unfortunately, there is very little quality control on the information disseminated to the masses via web‐enabled platforms, and people at all ages are vulnerable to receive misinformation rather than reliable, science‐based information. As such, universities and other conservation organizations have a critical role in developing a strong presence in podcasting and other online learning resources to provide reliable, science‐based information to students and stakeholders interested biological conservation.

Although the traditional forms of in‐person educational program delivery will continue to play an important role in the classroom and in public outreach, podcasts are poised to be one of the most effective forms of non‐traditional education because information can be effectively distributed to global audiences without the need for in‐person contact. Podcasts have a similar niche to webpages in that you only must build it once and can reach anyone, anywhere as long as they can connect to the internet. Podcasts will not supplant other forms of information delivery, but represent another tool to augment those other forms, and may reach new audiences not engaged by traditional forms of face‐to‐face information delivery (Hendrickson et al. 2010; Zobrist & Rozance, 2015). Also, they may bridge the gap for science delivery to audiences that do not have access to more formal delivery forms such as attending college.

## 2 DEER UNIVERSITY PODCAST AS A CASE STUDY

We provide an example using the podcast *Deer University* (https://deeruniversity.libsyn.com/) to demonstrate the potential of this emerging educational platform to reach audiences on conservation issues. White‐tailed deer (*Odocoileus virginianus*) are one of the most economically and ecologically important wildlife species in North America. In 2016, 8.1 million hunters spent 115 million days hunting deer, more than any other game species (USFWS, 2016). Deer hunters are a passionate group and are constantly seeking advice from natural resource professionals to improve deer habitat, deer numbers, and deer quality on their property. This provides a great opportunity to disseminate deer biology and management information that is research‐based, and not biased by product promotion. Moreover, private landowners, at least in some parts of the United States, are responsible for a large portion of natural lands and thus provide a critical role in biological conservation. Those same landowners often have a game species interests and often make habitat management decision based on those interests. Thus, focusing messaging on a species like deer that have a wide interest potentially can serve as a conduit to promote more holistic conservation practices on the landscape.

The *Deer University* podcast was launched in May of 2017 with the goal of providing answers to the questions most often asked by deer hunters and managers. The *Deer University* podcast compliments more traditional forms of public outreach by teaching concepts that are regularly featured during in‐person events, outreach publications, popular magazine articles, and email questions. The motto of the podcast was to take research‐based information and distill it in a way the layperson can understand and apply it. The podcast also provides a venue to discuss current issues that are often misunderstood by the general public, and in particular, deer hunters. Podcast episodes related to issues like Chronic Wasting Disease, the effectiveness of culling to improve antlers and genetics, supplemental feeding, habitat management, and USDA Natural Resources Conservation Service Programs, provide science‐based accurate information not easily found in hunting magazines, forums, and websites. Moreover, the podcast regularly featured topics related to fostering biodiversity and enhancing ecosystem services, albeit with a deer‐centric spin.

**FIGURE 2:**
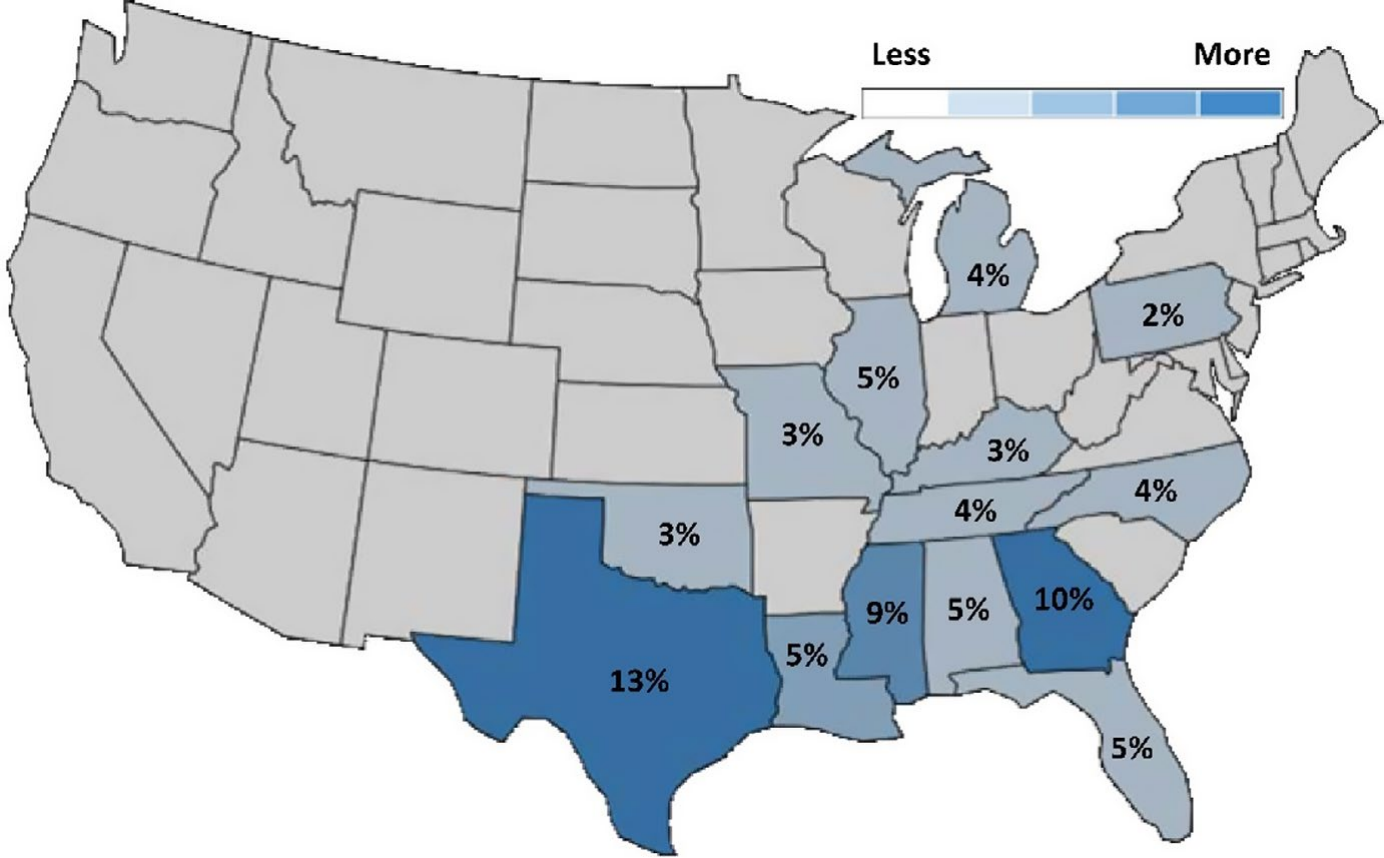
Map of the lower 48 US states showing the most common states the *Deer University* podcast is downloaded. The map was produced by the podcast hosting software libsyn (https://libsyn.com/). Numbers represent the percentage of total US downloads

Since launching the *Deer University* podcast in May 2017, the response has been overwhelmingly positive with content downloaded >265,000 times. The podcast has been downloaded in every US state, and in 25 countries—demonstrating the reach of this educational delivery method (Figure 2). Currently, there are 40 episodes with an average of 5,407 (range 2,510–15,920) downloads per episode. The feedback from listeners indicates that they like the podcast because it is based objectively on science and is not used for marketing products, as is commonplace in other information delivery forums particularly as they relate to this species.

## 3 USING PODCASTING FOR CONSERVATION EDUCATION

### 3.1 Developing a podcast

**FIGURE 3:**
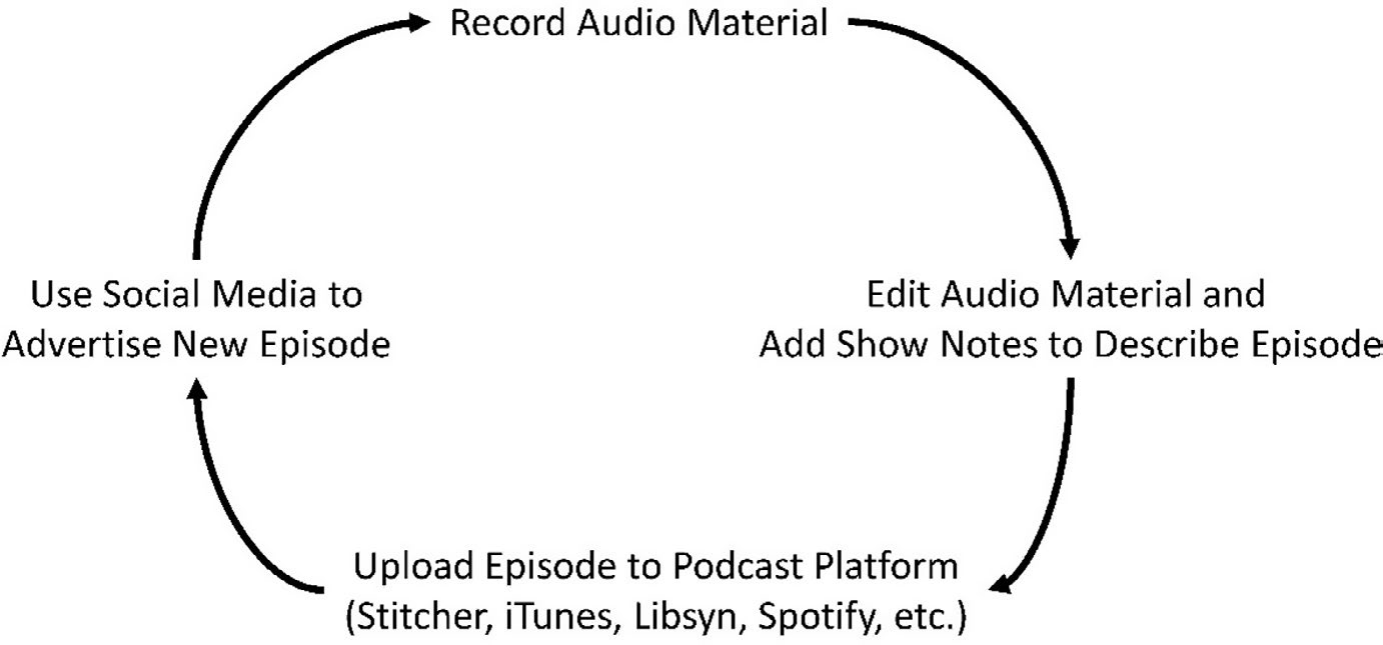
Steps for recording a podcast to disseminate educational material

Developing a podcast is relatively easy and inexpensive. Most of the audio editing and mixing software is available free online (e.g., Audacity for PC and Mac, GarageBand for Mac), but more powerful software is available for purchase. A quality microphone to record audio is very important and will likely be the largest expenditure (assuming you already have a computer). Good microphones can be purchased for less than $200 US dollars. Portable recording devices can also be used if a computer is not available or convenient to use (e.g., recording a podcast in the field). Podcasters can also use free online programs such as Zoom and Skype to record both audio and video of conversations to be used for a podcast. The process of podcasting is relatively easy (Figure 3). Once the audio has been recorded, the next step is to edit any distracting noises, or possibly edit the episode to meet a time constraint, and then add any pre‐recorded introductions or outgoing messaging (e.g., audacity, garageband, iMovie). The completed audio file is then uploaded along with the “show notes” that describes the content of an episode to a podcast platform (e.g., iTunes, Stitcher, Google Play, Libsyn, Podbean, etc.) where the episode is available for download. Some platforms are better than others in terms of linking episodes to multiple platforms, so be sure to check on this capability.

Platforms track download metrics differently. A platform that tracks download numbers as well as spatial information, download devices, etc., can be very useful when quantifying the reach and potential impacts of the podcast. Most platforms require a monthly fee to host a podcast and store the episodes. The monthly fee is related to the number of episodes you upload and storage you require. A couple of additional recommendations are (a) be sure to brand and market your podcast. As mentioned previously, there are greater than 1 million podcasts available to listeners so be sure the title of your podcast generally conveys the content to attract your target audience. (b) Be prepared to market your podcast with social media (see https://www.facebook.com/msu.deerlab/ and https://www.facebook.com/uf.deerlab/ for examples). Listeners can certainly find your podcast by actively searching for content, but you can greatly accelerate the growth of your podcast by advertising episodes via social media. (c) Relax and record a conversation. You do not need a detailed outline or a transcript, rather, prepare to discuss the meaningful topics and let the conversation meander just as it would if you were having the conversation with a colleague over lunch. This format makes the content much more accessible to people that are not in your field of science and listeners of *Deer University* commonly send comments to this effect. One other approach we are taking is to link multiple podcast labels together in a network. We have linked *Deer University* with *Fire University* (https://fireuniversity.libsyn.com/), *Habitat University* (https://habitatuniversity.libsyn.com/), and *Pond University* (https://ponduniversity.libsyn.com/) into a single umbrella network called *Natural Resource University* (https://naturalresourcesuniversity.libsyn.com/). The idea being that the network can create synergy between podcasts and associated social media platforms to leverage the following of each individual podcast to more effectively target information to relevant constituents of others. This collaborative approach is particularly effective at rapidly building a following upon launch of a new podcast. We tested this idea with the *Natural Resources University* network and found that each episode garnered more than twice as many downloads when combined into the network than the same episode released individually through the respective podcasts platform individually.

### 3.2 Podcasting for public outreach

In the United States, the Cooperative Extension Service at land grant universities is a product of the Smith‐Lever Act of 1914. The goal the Extension Service is taking research‐based information from universities and presenting the information in a way to inform citizens about issues of interest. This same goal is accomplished in other countries but is called other terms such as public outreach and stakeholders knowledge sharing. Broad areas of this public outreach programming include, but are not limited to, agriculture, range and forest management, fish and wildlife management, health and well‐being, and youth education. Historically, teaching events for public outreach professionals have included field days, workshops, and seminars, but now include websites, blogs, videos, social media, and podcasting. Podcasts provide an opportunity to reach an audience at scales that would never be possible with traditional face‐to‐face teaching events. Another major advantage of podcasts over that of other more traditional forms of public outreach is that they can be recorded quickly to rapidly respond to dramatic events such as natural disasters, disease outbreaks, or changes in legislation. Although podcasting is in its infancy within public outreach programming, this is certainly an emerging education tool that will only grow in popularity over time.

### 3.3 Podcasting in the classroom

While podcasting is widely used as a tool to enhance pedagogical approaches in the classroom (Beamish & Brown, 2008; Flanagan & Calandra, 2005; Frydenberg, 2006; Lazzari, 2007), it is not widely used in ecology and evolutionary biology classrooms. Given that podcasts are digital, highly versatile in terms of length and content, and are offered asynchronously, they provide a viable tool to safely deliver content to students in the wake of the pandemic and may be robust to changes in society over time. Lashley and McCleery (2020) suggested podcasting could be used to mix up content in a flipped classroom format, provide an opportunity to interview experts on a given topic, and even provide an opportunity for students to engage in the process of developing and producing content. They suggested short 5–15‐min podcast episodes could be an effective length to engage students attention, provide an opportunity to review the content, and because it is digital, could be easily shared between instructors covering similar topics to increase the availability and diversity of content. Also, we included one podcast in Table 1, *How ecology works*, which is structured according to the recommendations in Lashley and McCleery (2020) which includes ~15‐min episodes on a wide range of ecology topics designed specifically to be used in ecological college courses. We believe incorporating podcasts, whether recorded by the instructor or from a freely available repository such as *How ecology works*, into the classroom could be a good way to enhance the online learning experience.

**TABLE 1:** Science‐related podcasts suggested by Ecologists and Evolutionary biologists replying to a post on Ecolog (a list serve hosted by the Ecological Society of America) and on our social media pages^a^

Podcast title	Affiliation or Host^b^	Brief summary of subject area^c^
Acres U.S.A.: Tractor Time	Ben Trollinger	Discusses ecological techniques to improve agricultural production while decreasing inputs.
AgMatters	Mississippi State University Extension Service	Why agriculture matters.
Agricultural Economics Podcast	Purdue University	Recent news on agricultural economics issues.
Big Biology	Marty Martin, Art Woods	Interviews with leading scientists about the biggest unanswered questions in biology, especially related to evolutionary theory.
Bioscience Talks	American Institute of Biological Sciences	Features in depth discussions of recently published Bioscience articles.
Bird Note Podcast	BirdNote	Focused on delivering information about birds to a general audience.
Building a Vibrant Community	Mississippi State University Extension Service	Community development problems and how to make communities grow.
Blue Earth	Future Frogmen	Water‐related topics in the context of biological conservation.
British Ecological Society Journals	British Ecological Society	Covering the latest research in ecology, especially research publish in BES journals.
Camp 8	University of Minnesota Extension	Information from foresters, wildlife managers, and researchers.
Coffee and Conservation	Mississippi State University Extension Service	How to apply conservation to support productivity and profitability.
Conservation Matters	Shane Mahoney	Explores history and philosophy of wildlife conservation and management, especially the North American model of Wildlife Conservation.
Crime Pays But Botany Doesn't		Explores topics related to botany and ecology for a general audience.
Deer University	Mississippi State University Extension Service	Drs. Bronson Strickland and Steve Demarais discuss the science behind white‐tailed deer ecology and management and interview deer‐related researchers. Part of Natural Resources University podcast network.
Discovering Darwin	Sarah Bray, James Wagner	Discusses theory of evolution, mechanisms, and historical and contemporary‐related research.
Dr. Tim's Spineless Wonders	Purdue University	Real‐life insect encounters and other entomology stories.
Earth and Environmental Sciences	Colorado School of Mines	Topics typically span the earth sciences.
EcoBeneficial! Podcast	Ecobeneficial!	Kim Eirman interviews leading experts on environmental topics with a focus on sustainable practices for a general audience.
Ecological Adventures	University of Florida, IFAS	Discusses experiences of the faculty and students of the Department of Wildlife Ecology and Conservation, University of Florida.
Ecology in your Face	University of Florida, IFAS	Dr. Marcus Lashley and Charlotte Nowak interview scientists about the most interesting, exciting, and timely ecological topics. The breadth of topics spans all ecosystems and all corners of the globe.
Ecology is Everywhere	The ecology school at River Bend	Covers topics related to ecology and environmental sciences geared toward pre‐college aged students.
Everything Hertz	James Heathers, Dan Quintana	The two scientists discuss issues related to academia and the scientific process in general.
Evolution 101	Zachary Moore	Focuses on answering listener questions and describing evolutionary concepts in layman's terms.
Evolution Talk		A podcast focused on discussing the mechanisms of evolution for a general audience.
Evolution, Ecology, and Behavior – Audio	Yale University	Presented as a course in the principles of evolution, ecology, and behavior for students beginning college in EEB.
Field Crops IPM	University of Minnesota Extension	Emerging field crop pest concerns and pest management.
Fire University	University of Florida, IFAS	Dr. Marcus Lashley interviews scientist and professionals on issues related to natural resource management with particular focus on fire and wildlife ecology. Part of Natural Resources University podcast network.
Future Ecologies	Adam Huggins, Mendel Skulski	Storytelling about the way humans relate to the earth featuring interviews of scientist in each episode.
Good Life Revival	Sam Sycamore	Focused on how to be more sustainable, and covers many topics related to environmental sustainability.
Gopher Coffee Shop	University of Minnesota Extension	Crop production and related issues.
Got Nature?	Purdue University	Broad range of topics on forestry, wildlife, and aquatic sciences.
Green Dreamer	Kamea Chayne	Covers a broad array of topics related to environmental sustainability.
Habitat University	Purdue University Extension	Jared Brooke interviews scientists and professionals on issues related to wildlife habitat management.
Hot and Dry Podcast	The Southwest Fire Science Consortium	Focuses on issues and related to climate change in the Southwestern U.S.A. and strategies to mitigate those effects.
How ecology works	University of Florida, IFAS	Short interviews of expert scientists on a broad array of ecological concepts and applications designed specifically to be used as supplemental material in ecological‐focused undergraduate college courses.
How to save the world		Jaimie and Meghan discuss topics related to how to be more sustainable, particularly for general audiences during everyday activities.
HumaNature	Wyoming Public Media	Stories about real experiences in the natural world from people's adventures.
In Defense of Plants		Covers topics related to the unique wonders of plants.
Introduced	Wisconsin Sea Grant	Stories related to nonnative aquatic species invasions, especially in the Great Lakes region of the U.S.A.
Land and Legacy Podcast		Focuses on the planning and application of habitat management practices specifically for game species on private lands in the U.S.A.
Major Revisions	Jeff Atkins, Jon Walter, Grace Wilkinson	Hosted by three early career environmental scientists; topics focused on ecology and academia in general.
Making Waves	Society of Freshwater Science	Interviews of scientist on the current freshwater‐related research. Topics include biodiversity, ecology, and technology
Meateater	Steven Rinella	A podcast about hunting but regularly features interviews of wildlife biologist and ecologists about the science of wildlife conservation.
Mongabay Newscast	Mongabay	Interviews practitioners in natural science fields and discusses global environmental issues.
Natural Resources University	Iowa State University, Mississippi State University, Purdue University, University of Florida	Network of science‐based podcasts focused on delivering information related to the conservation of natural resources to scientist, practitioners, and the general public.
Naturally Speaking	University of Glasgow	Interviews scientists covering a wide range of ecological and environmental topic areas.
Nature Check	Sheryl Hosler	A themed game world approach to discuss issues related to biology, ecology, environmental science, research, and society.
Northwest Nature Matters	Oregon Wildlife Foundation	John Goodell discusses a variety of ecological topics especially wildlife ecology in the northwestern U.S.A.
Nutrient Management Podcast	University of Minnesota Extension	Nutrient management for crops and best management practices.
Ocean Science Radio	Naomi Frances Farabaugh, Andrew Kornblatt	Highlights the latest ocean science stories.
Ologies	Allie Ward	Intentionally focuses on a broad array of topics but mostly within STEM disciplines.
Outside/In	New Hampshire Public Radio	Covers a broad spectrum of environmental issues targeting a general audience.
Overheard	National Geographic	Broadly explores nature across disciplines and presented for a general audience.
Palaeocast		Explores the fossil record and the evolution of life on earth.
Planted: Finding your roots in STEM Careers	The Morton Arboretum	Interviews professionals that work with plants with the intent to introduce students and early career scientists to the job opportunities in the field.
Plasticast	Pacific Northwest Consortium	Topics related to microplastics and environmental toxicology.
PolliNation	Oregon State University Extension	Tells stories of professionals and citizens who striving to improve pollinator health.
Pond University	Purdue University Extension	Dr. Mitchell Zischke discusses the latest research and management strategies for management of freshwater fisheries. Part of Natural Resources University podcast network.
Purdue Commercial AgCast	Purdue University	Farm management news and advice.
Regenerative Agriculture	John Kempf	Ecology and economics in agricultural landscapes.
Science Diction	Johanna Mayer	Discusses the origin of words and the science stories behind them.
Science Friday	Ira Flatow	Discusses news about science and technology.
Science VS	Gimlet	Explores the science behind topics of general interest to the nonscientist public.
Soybean Pest Podcast	Iowa State University Extension and Outreach	Drs. Matt O'Neal and Erin Hodgson discuss the ecology and evolution of insect pests, management strategies, and other related topics in agriculture.
Smart Gardens	University of Minnesota Extension	Questions answered about gardening and outdoor living.
Strictlyfishwrap Science Radio Hour	WRFR‐LP Community Radio Station	Skyler Bayer interviews guests about science in Maine, U.S.A.
Subsurface	Montana Public Radio	Focused on invasive species, especially those affecting aquatic systems in Montana U.S.A.
Superheroes of Science	Purdue University	Information on current science and latest trends in science communication.
Sustainable World Radio		Interviews experts who study and work in nature and focuses on sustainable food production and sustainable use of natural resources.
Swine Podcast	University of Minnesota Extension	Discussion of swine‐related research topics.
Teach me about the great lakes	Stuart Carlton, Illinois‐Indiana Sea Grant	Interviews professionals about natural resource topics in the great lakes region in North America.
Tetrapod Zoology podcast		Discussion related to amphibians, reptiles, birds, and mammals in a wide variety of contexts.
The Common Descent Podcast	Will Harris, David Moscato	Covers a wide variety of evolution‐related topics and paleontology with discussions about biological diversity.
The Crop Situation	Mississippi State University Extension Service	Science‐based information about agricultural row crops.
The EcoCiv Podcast	Institute for Ecological Civilization	Covers a wide variety of topics related to society and ecology.
The Fisheries Podcast	Brett Kelly, Nick Kramer, Julie Vechio	Focuses on telling the story of people and projects in the fisheries discipline.
The Graduates Sci‐Com	Trinity Livingston	Explains science in a fun way primarily by interviewing graduate students about their research. Topics cover broad areas but tend to be related to ecology.
The Hunter Conservationist	Mark Hall	Discusses hunting adventures, interviews wildlife biologists, and reflects on wildlife and habitat conservation.
The Moos Room	University of Minnesota Extension	Topics for beef and dairy producers.
The Mountain Nature and Culture Podcast		Broadly discusses a variety of topics but covers wildlife ecology topics frequently.
The Natural Selection	University of Exeter's Centre for Ecology and Conservation	Communicates biological research from the university to the general public.
The Story Collider		Interviews professionals across disciplines to deliver science information to a general audience.
The Thriving Farmer	Michael Kilpatrick	Discusses a wide array of topics related to managing natural resources in agriculture for producers.
The Wild Podcast	Chris Morgan	A bear biologist explores the complexities of ecosystems with a focus on the pacific northwest in the USA.
This podcast will kill you	Erin Allmann Updyke, Erin Welsh	Erin and Erin discuss disease ecology and medical mysteries.
This week in Evolution	Nels Elde, Vincent Racaniello	Focuses on a variety of topics related to evolution focuses on genomics and molecular biology.
Today's Voices of Conservation Science	Depart. Of Ecology, Montana State University	Andrea Litt and Chris Guy interview professionals that use science to conserve natural resources and discuss their paths to get there.
Undiscovered	Elah Feder, Annie Minoff	Discusses seemingly random events that lead to innovation in science.
Vital Connection On Air	University of Minnesota Extension	Trends and topics important to MN communities and leaders.
What's Killing My Kale?	University of Minnesota Extension	Pest issues affecting fruit and vegetable crops.
Wild Animals	North Carolina Museum of Natural Sciences	Biologists are interviewed about their experiences working with wildlife with a focus on unique individuals they have encountered.
Wild Roots		Discusses the relationship of humans with nature in a variety of contexts.
Youth Development Podcast	University of Minnesota Extension	Youth workers discuss research, theory, and best practices.
4‐H‐4‐U‐2	Mississippi State University Extension Service	Mississippi 4‐H youth programs and positive youth development.

^a^ This table is strictly reporting podcasts labels recommended by our peers on Ecolog and from social media followers of the authors and thus, does not indicate an endorsement of the podcast or content. We reported all titles recommended and excluded none. Therefore, the list is US centric but included any recommended podcasts from other countries.

^b^ The affiliation was listed when an organization was hosting the podcast and otherwise the host and cohosts. It was left blank in the case that neither information was readily apparent on the podcast website.

^c^ The subject area was paraphrased based on information from the “about” section on the website, from the person who submitted the label, or from a brief sampling of the available episodes and thus, may not fully accurately describe the contents of the podcast.

### 3.4 Finding relevant podcasts

Finding relevant podcasts to target your specific needs can be a challenge. In an effort to facilitate the locating of relevant podcasts, we compiled a list of science‐relevant podcast labels (Table 1). We compiled this list by asking the ecology and evolution community through each author's social media networks and through a post on ecology, a listserv hosted by the Ecological Society of America, to respond with their favorite, or their own, podcast that covered an ecology or evolutionary biology‐related subject area. We received responses from 128 individuals with more than 70 unique podcast suggestions. This list is not exhaustive and was not intended to exclude or endorse any particular label, but instead, we hope that it provides a good starting point for finding podcasts relevant to individual needs based on recommendations from our community. We recognize that this list by nature of the way we compiled it is strongly biased to North America and there are likely many science‐based podcasts available related to this topic in languages other than English and in other parts of the globe.

## 4 PANDEMIC PODCASTING

The COVID‐19 pandemic has, hopefully only temporarily, eliminated or modified traditional classroom teaching at universities (Crawford et al., 2020). These changes have had an immediate and dramatic impact on ecology and evolution‐related disciplines (Corlett et al., 2020) and may lead to a long‐term shift on the reliance of remotely delivered communications (Lashley et al., 2020). Podcasting is an underutilized tool that can be used to directly and safely to teach students during the pandemic, and this technology can additionally be used to educate a far greater number of people in broader audiences such as those targeted through public outreach programming. Because podcasts can be recorded and delivered to broad audiences remotely with technologies already available, we believe this platform provides a useful tool in the pedagogical platform for science education.

## 5 CLOSING REMARKS

Podcasting is an emerging tool with a broad flexibility to deliver science‐based information asynchronously in the online classroom or in online outreach programming. This platform provides an additional opportunity for scientist to convey science‐based information directly from scientist to a broad range of end users globally in near real time. Because this technology is being widely adopted across demographics, using this tool for online learning in ecology and evolution is likely to improve communication skills in our disciplines in a safe and inclusive manner. Thus, we encourage ecologist and evolutionary biologists to consider adopting podcasting as part of their programs.

## CONFLICT OF INTEREST

None declared.

## AUTHOR CONTRIBUTIONS

**Bronson T. Strickland:** Conceptualization (equal); Funding acquisition (equal); Methodology (equal); Project administration (lead); Resources (equal); Writing‐original draft (lead); Writing‐review & editing (equal). **Jarred M. Brooke:** Conceptualization (supporting); Funding acquisition (supporting); Project administration (supporting); Writing‐review & editing (supporting). **Mitchell T. Zischke:** Conceptualization (supporting); Funding acquisition (supporting); Project administration (supporting); Writing‐review & editing (supporting). **Marcus A. Lashley:** Conceptualization (equal); Funding acquisition (equal); Project administration (equal); Resources (equal); Writing‐original draft (equal); Writing‐review & editing (equal).

## ETHICAL APPROVAL

No use of animal or human subjects in this study.

## Data Availability

Data used to create graphs are archived at the given web address and freely available for download. All data collected to produce the table are included within the table in this manuscript.
